# Pharmacologically reversible, loss of function mutations in the TM2 and TM4 inner pore helices of TREK-1 K2P channels

**DOI:** 10.1038/s41598-019-48855-1

**Published:** 2019-08-27

**Authors:** Ehab Al-Moubarak, Emma L. Veale, Alistair Mathie

**Affiliations:** Medway School of Pharmacy, University of Kent, Central Avenue, Chatham Maritime, Kent, ME4 4TB UK

**Keywords:** Ion channels in the nervous system, Molecular neuroscience

## Abstract

A better understanding of the gating of TREK two pore domain potassium (K2P) channels and their activation by compounds such as the negatively charged activator, flufenamic acid (FFA) is critical in the search for more potent and selective activators of these channels. Currents through wild-type and mutated human K2P channels expressed in tsA201 cells were measured using whole-cell patch-clamp recordings in the presence and absence of FFA. Mutation of the TM2.6 residue of TREK-1 to a phenylalanine (G171F) and a similar mutation of TM4.6 (A286F) substantially reduced current through TREK-1 channels. In complementary experiments, replacing the natural F residues at the equivalent position in TRESK channels, significantly enhanced current. Known, gain of function mutations of TREK-1 (G137I, Y284A) recovered current through these mutated channels. This reduction in current could be also be reversed pharmacologically, by FFA. However, an appropriate length MTS (MethaneThioSulfonate) cross-linking reagent (MTS14) restricted the activation of TREK-1_A286C channels by repeated application of FFA. This suggests that the cross-linker stabilises the channel in a conformation which blunts FFA activation. Pharmacologically reversible mutations of TREK channels will help to clarify the importance of these channels in pathophysiological conditions such as pain and depression.

## Introduction

TREK-1 (TWIK-related K, also K_2P_2.1, *KCNK2*) potassium channels are members of the two pore domain (K2P) channel family^1–3^ and contribute to background potassium conductances in many cell types. Their activity can be regulated by a variety of physical and chemical stimuli^4,5^. TREK-1 channels have many structural and functional properties in common with two closely related K2P channels, TREK-2 and TRAAK. Indeed, evidence suggests that all three channels can form functional heterodimeric channels with each other^6–8^. Collectively, these three channels can be grouped together as the “TREK” subfamily of mechano-sensitive K2P channels^1^.

There is increasing evidence in support of a role of TREK channels in nociceptive processing, which may offer hope of new therapeutic advances in the treatment of pain (e.g.^9,10^). Fenamate compounds, such as flufenamic acid (FFA), are non-steroidal anti-inflammatory drugs (NSAIDs) used clinically in the treatment of pain. These compounds have been shown to up regulate the activity of TREK channels^11,12^. BL-1249, another fenamate-like structure and a putative activator of TREK-1-like currents in human bladder myocytes^13^ has also been shown to activate TREK-1 and TREK-2 channels directly^12,14–16^.

FFA and other fenamate-like compounds interact with a directly accessible binding site^12,14^, since FFA is effective in inside-out patch recordings and BL-1249 enhances current through purified TREK-2 protein reconstituted into a lipid bilayer^14^. BL-1249 has been shown to bind to a negatively charged activator site (NCA) to open many K2P channels through gating at the selectivity filter^15^. Fenamates do not bind to the cryptic binding site at the selectivity filter identified by Lolicato *et al*.^17^ for the activators ML335 and ML402^15–17^.

All mammalian K2P channels possess a key “hinge” glycine (G) residue in transmembrane domains TM2 and TM4, the inner pore helices of the channel, which provide flexibility at the centre of these helices. For the TM2 helix, another important residue has been identified five amino acids further towards the C terminus end of the channel after these hinge G residues, termed TM2.6, with the hinge G residue defined as TM2.1^18^. This amino acid varies across the family of K2P channels, but is primarily a hydrophobic leucine residue when considering both vertebrate and invertebrate channels. Mutation of this TM2.6 residue to an aspartate or asparagine in all K2P channels studied, produces a gain of function channel^18^. These mutations cause substantial increases in channel open probability (Po) with no substantive change in single channel conductance or the amount of channel expressed at the cell membrane. This has been proposed to give a simple and powerful strategy to systematically manipulate activity of the entire K2P channel family^18^.

In this study, we show that mutation of the TM2.6 of TREK-1 to a phenylalanine residue (G171F) and a similar mutation of TM4.6 (A286F) substantially *reduces* current through TREK-1 channels and, importantly, that this reduction in current can be reversed, pharmacologically, by FFA.

## Results

### The G171F mutation at position TM2.6 reduces TREK-1 current

**Figure 1:**
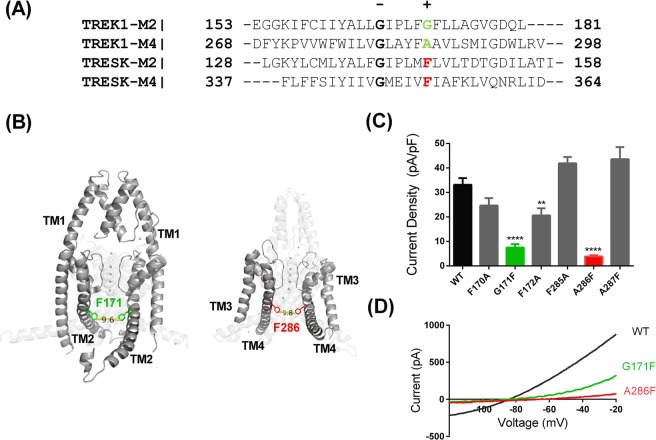
Loss of function through mutation of the TM2.6 and TM4.6 inner pore residues of TREK-1 channels. (**A**) Alignment of the inner pore amino acids of TREK-1 and TRESK channels. The conserved hinge glycine residues are indicated by “−” and the TM2.6 and TM4.6 amino acids by “+”. (**B**) Illustration of hTREK-1_G171F/A286F structure created from the pdb file for the crystal structure of TREK-1 (6GC6)^17^. For clarity, TM3 and TM4 are rotated through 90° and separated from TM1 and TM2. F171 residues are green, F286 residues are red. (**C**) Histogram of outward currents measured as the difference current between that at −40 mV and −80 mV for WT TREK-1 and mutated TREK-1 channels (** = significantly different from wild type at <0.05 level, **** = significantly different from wild type at <0.001 level, 1-way ANOVA followed by Dunnett’s test, WT vs F172A, p = 0.0007; WT vs G171F, p < 0.0001; WT vs A286F, p < 0.0001). (**D**) Representative current-voltage relationships for WT TREK-1, TREK-1_G171F and TREK-1_A286F channels.

The presence of a glycine residue at TM2.6 in TREK channels is consistent with what is seen for most non-K2P potassium channels^19^. However, somewhat unusually, the non-selective cation NaK channel from *Bacillus cereus* has a bulky phenylalanine (F) residue at the equivalent position and a concomitant small ion flux through the channel^20^. The mammalian K2P channel TRESK also has F at this position (Fig. 1A). We therefore mutated the TM2.6 glycine residue in TREK-1 (Fig. 1A) to phenylalanine (G171F). A TREK-1 crystal structure^17^ (6GC6, see Fig. 1B) confirms that the G171 side chain at TM2.6 is at the intracellular side of TM2 exposed to a large cytoplasmic vestibule and in close proximity to the intracellular face of the selectivity filter. This G171F mutation significantly reduced the TREK-1 current from 33.2 ± 2.7 pA/pF (mean ± SEM, n = 16) for WT TREK-1 to 7.5 ± 1.3 pA/pF (n = 17) a reduction of 77% (Fig. 1C,D). Mutation of amino acids at positions TM2.5 and TM2.7 on either side of G171 in TM2 (F170A, F172A) had much less effect on current amplitude (Fig. 1C), giving a modest reduction in current compared to that seen for G171F. Thus, TM2.6 mutations can either increase^18^ or decrease the activity of mechano-gated K2Ps, depending on the substitute amino acid chosen.

### The A286F mutation at position TM4.6 reduces TREK-1 current

Because K2P channels have two pore domains and form dimers, the TM2 region of the channel is opposed to the TM4 region. The TM4 region of K2P channels can be classified in the same way as TM2, with the hinge G residue defined as TM4.1. The TM4.6 residue in K2P channels is more variable than the TM2.6 residue across species, with the most common residues being threonine, serine or alanine^18^.

It was of interest to determine whether the same phenylalanine mutation at the equivalent site on the TM4 region of TREK-1 channel (TM4.6) had similar effects on channel current to that seen for the TM2.6 mutation. The A286F mutation significantly reduced the TREK-1 current from 33.2 ± 2.7 pA/pF (mean ± SEM, n = 16) for WT TREK-1 to 3.8 ± 0.5 pA/pF (n = 8), a reduction of 89% (Fig. 1C,D). Mutation of amino acids at positions TM4.5 and TM4.7 on either side of A286 in TM4 (F285A, A287F) had no effect on current compared to WT (Fig. 1C).

### Mutations at positions TM2.6 and TM4.6 enhance current through TRESK channels

**Figure 2:**
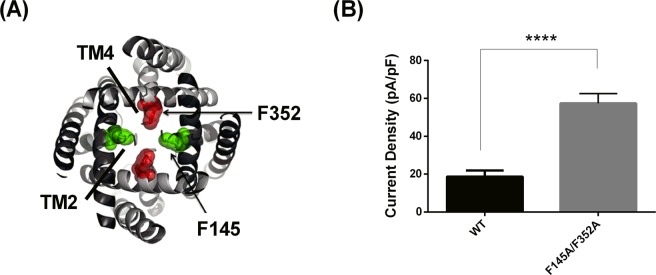
Gain of function through mutation of the TM2.6 and TM4.6 inner pore residues of TRESK channels. (**A**) Model of hTRESK based on TRAAK crystal structure, highlighting the constriction point amino acids in the inner pore. F145 residues are green, F352 are red. (**B**) Histogram of outward current measured as the difference current between that at −40 mV and −80 mV for WT TRESK and TRESK_F145A/F352A channels (****p < 0.001, t-test).

For TRESK channels, the TM2.6 and TM4.6 residues are, uniquely for mammalian K2P channels, phenylalanine residues (F145, F352). An homology model of TRESK suggests that these amino acids are also exposed to a large cytoplasmic vestibule (see Fig. 2A). Alteration of these residues to less-bulky alanine residues, a F145A_F352A double mutation, significantly increased TRESK current from 18.7 ± 3.2 pA/pF (n = 7) for WT TRESK to 57.4 ± 5.2 pA/pF (n = 6) for the F145A_F352A double mutation, an increase of 207% (Fig. 2B), complementary to what was observed for the reverse TREK-1 channel mutations at TM2.6 and TM4.6.

### Flufenamic Acid (FFA) as a modulator of TREK-1 channel gating

**Figure 3:**
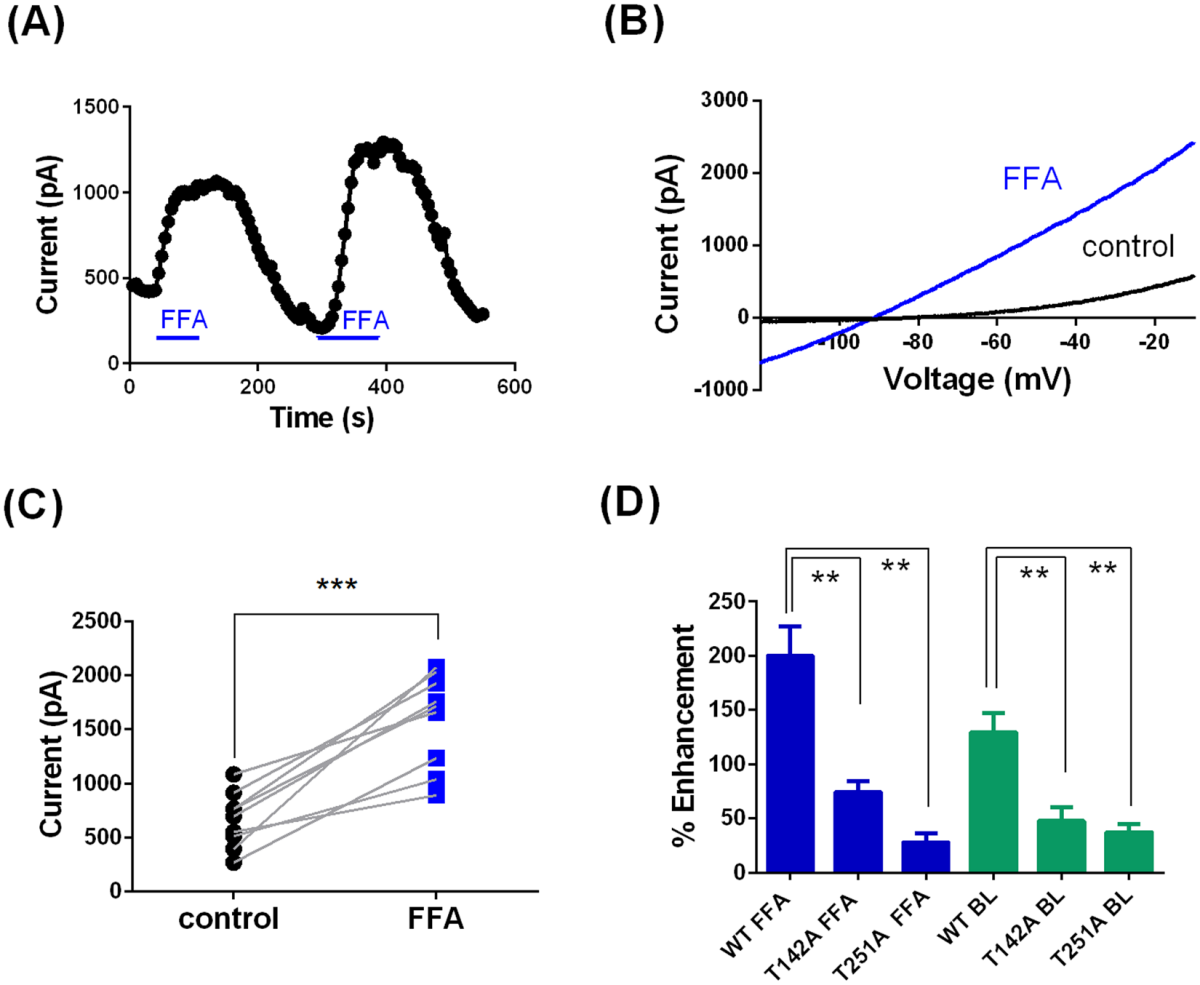
FFA enhancement of TREK-1 channel current is blocked by selectivity filter mutations. (**A**) Representative time course for enhancement of WT TREK-1 current by FFA (100 µM). Application of FFA is indicated by the blue bar. (**B**) Representative current-voltage relationship for WT TREK-1 channels in the presence (blue) and absence of FFA. (**C**) Enhancement of WT TREK-1 current by FFA in individual cells (*** = significant difference following application of FFA at <0.01 level, paired t test). (**D**) Histogram of percentage enhancement by FFA (100 µM, blue) and BL-1249 (1 µM, green) of WT TREK-1 and mutated TREK-1 channels (** = significantly different from wild type at <0.05 level, ANOVA followed by Tukey’s test).

FFA (100 µM) significantly enhanced WT TREK-1 channels by 200 ± 27% (n = 18, Fig. 3A–C), consistent with previous observations^12^. The current increase was rapid and reversible when the drug was washed off. The FFA effect was reproducible, and subsequent drug applications enhanced the TREK-1 current similarly.

**Figure 4:**
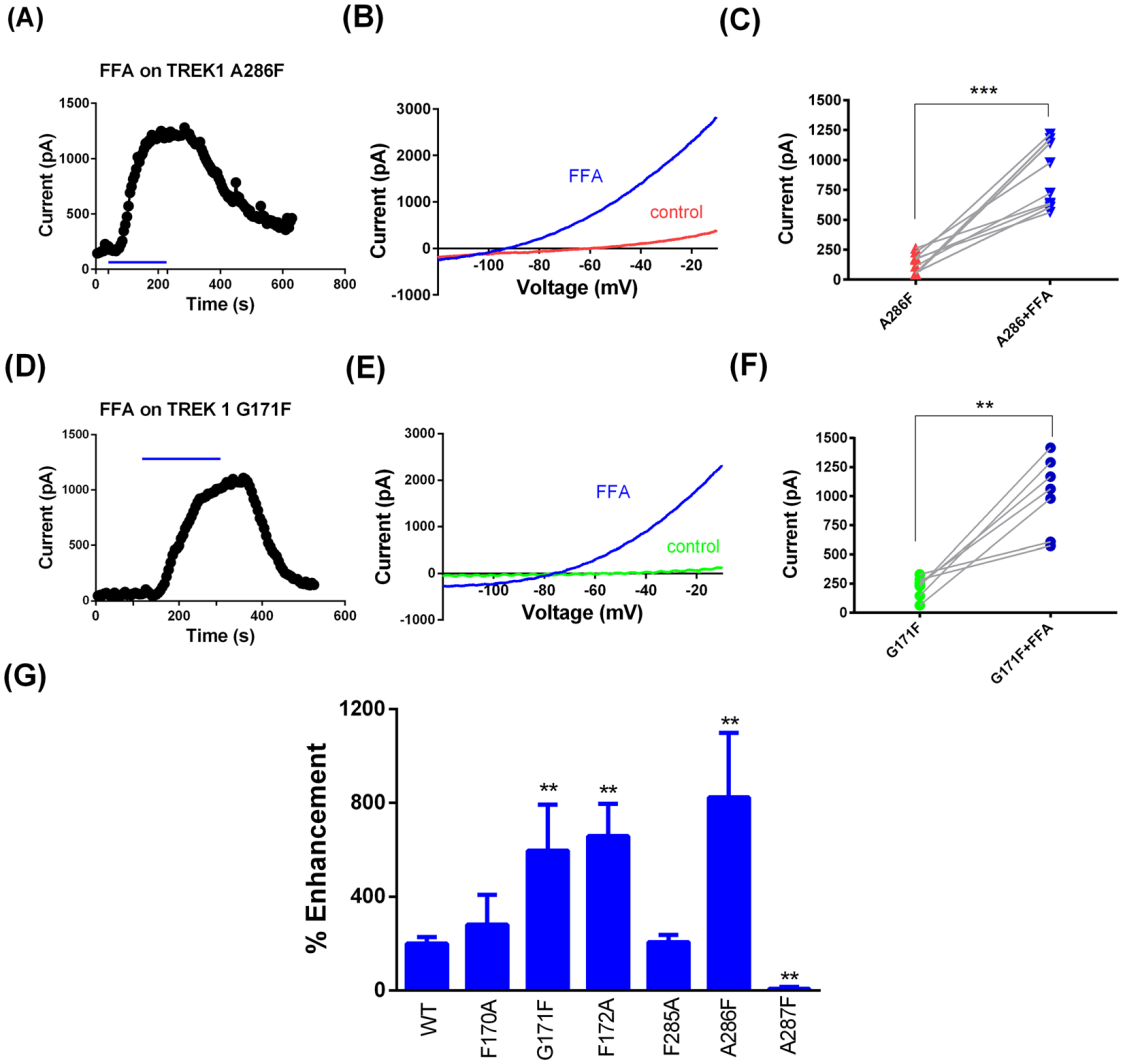
FFA substantially enhances current through TM2.6 (G171F) and TM4.6 (A286F) loss of function mutated TREK-1 channels. (**A**) Representative time course for enhancement of TREK-1_A286F current by FFA (100 µM). (**B**) Representative current-voltage relationship for TREK-1_A286F channels in the presence (blue) and absence (red) of FFA. (**C**) Enhancement of TREK-1_A286F current by FFA in individual cells (*** = significant difference following application of FFA at <0.01 level, paired t test). (**D**) Representative time course for enhancement of TREK-1_G171F current by FFA (100 µM). (**E**) Representative current-voltage relationship for TREK-1_G171F channels in the presence (blue) and absence (green) of FFA. (**F**) Enhancement of TREK-1_G171F current by FFA in individual cells (** = significant difference following application of FFA at <0.05 level, paired t test). (**G**) Histogram of percentage enhancement by FFA (100 µM) of WT TREK-1 and mutated TREK-1 channels (** = significantly different from wild type at <0.05 level, ANOVA followed by Tukey’s test).

The binding site for FFA is unlikely to be to the same site as that described for the blocking compound norfluoxetine on TREK-2^14^. Dong *et al*.^14^ identified a number of amino acids as “contact points” for norfluoxetine on TREK-2 including F316 and L320 in the TM4 region. Mutation of the equivalent amino acids in the TM4 region of TREK-1, F285A (see Fig. 4) and L289A had no effect on FFA-mediated enhancement of TREK-1, with the latter mutation showing 214 ± 85% (n = 7) enhancement of current by FFA (100 µM). This latter amino acid (L304 in the longer form of rat TREK-1 channels (NM_172042) used by Schewe *et al*.^15^) has been proposed to contribute to the binding site for BL-1249 on rat TREK-1 channels and mutation of this leucine does reduce the EC_50_ for BL-1249, to values closer to that seen for FFA^15^.

Schewe *et al*.^21^ have shown that TREK-1 and other K2P channels are gated by voltage through an “ion-flux” gating mechanism transduced at the channel selectivity filter (see also^22–25^). This gating is inhibited by mutation of the highly conserved threonine residues within the consensus K-selective motif (TIGFG) in both P1 and P2 (T142 and T251 in the isoform of TREK-1 used in this study). Mutations of these residues also inhibited enhancement of TREK-1 current by arachidonic acid and PIP_2_^21^. Notably, we have found that T142A (also T142C) and T251A mutations significantly reduced enhancement of TREK-1 current by both FFA and BL-1249 (Fig. 3D). This supports the suggestion that pharmacological enhancement of TREK-1 current by fenamates, like enhancement by arachidonic acid and PIP_2_, converges on this primary gate at the selectivity filter^15,16^.

### FFA recovers the blocking effects of the TM2.6 and TM4.6 loss of function mutations on TREK-1

Interestingly, FFA (100 µM) rapidly and reversibly enhanced TREK-1 A286F by 823 ± 275% (n = 9), Fig. 4A–C,G), and TREK-1 G171F by 523 ± 196% (n = 6), (Fig. 4D–F,G). Indeed, the current of these TREK-1 mutants, following FFA exposure, exceeded the average TREK-1 WT current.

### Adjacent F172A and A287F mutations alter the FFA effect on TREK-1

The effect of FFA on mutations of residues adjacent to TM2.6 and TM4.6, was more complex, despite the fact that these four mutations had modest or no effect on basal TREK-1 current size (see Fig. 1C). For TM2.5 and TM4.5 (F170A, F285A), enhancement of current by FFA was not significantly different to that seen for WT TREK-1 channels and so unaffected by the removal of a bulky residue from this position. However, mutation to remove the bulky residue at TM2.7 of TREK-1 (F172A) gave an enhancement by FFA of 658** ± **138% (n = 10), significantly greater than that seen for WT TREK-1 channels (Fig. 4G). This enhancement was reversible and reproducible. By contrast, the TM4.7 mutation (A287F) virtually abolished the FFA effect with an enhancement of just 7** ± **8% (n = 8, Fig. 4G) indicating that the introduction of a bulky F at TM4.7 nullifies the FFA activation of TREK-1. Thus the presence of a bulky residue at position TM*x*.7 either naturally (TM2.7) or through mutagenesis (TM4.7) reduces the effectiveness of FFA in activating TREK-1 channel current.

### pH_ext_ 8.4 does not recover the TM4.6 TREK-1 A286F current to WT current levels

**Figure 5:**
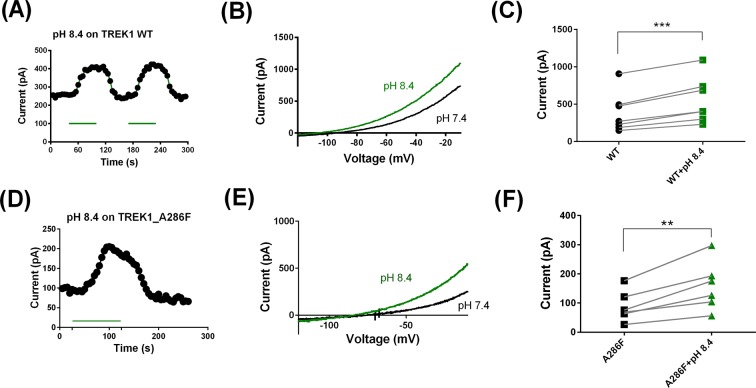
Alkalinisation has similar, modest effects on WT and TM4.6 (A286F) loss of function mutated TREK-1 channels. (**A**) Representative time course for enhancement of WT TREK-1 current by pH 8.4. (**B**) Representative current-voltage relationship for WT TREK-1 channels in pH 8.4 (green) and pH 7.4. (**C**) Enhancement of WT TREK-1 current by pH 8.4 in individual cells (*** = significant difference at <0.01 level, paired t test). (**D**) Representative time course for enhancement of TREK-1_A286F current by pH 8,4. (**E**) Representative current-voltage relationship for TREK-1_A286F channels in pH 8.4 (green) and pH 7.4. (**F**) Enhancement of TREK-1_A286F current by pH 8.4 in individual cells (** = significant difference at <0.05 level, paired t test).

Changes in extracellular pH alter TREK-1 and TREK-2 current by affecting structural elements at the channel’s extracellular terminus near and around the selectivity filter^26^. Raising extracellular pH, from 7.4 to 8.4, significantly enhanced WT TREK-1 current by 50 ± 6% (n = 7, Fig. 5A–C). Similarly, raising the extracellular pH, from 7.4 to 8.4, enhanced TREK-1 A286F by 84 ± 16% (n = 6, Fig. 5D–F). Unlike FFA, this pH change did not recover the TM4.6 loss of function TREK-1 channel current to WT TREK-1 current levels. Furthermore, the enhancement by pH 8.4 of TM4.6 loss of function TREK-1 channel current is not significantly more than that seen for WT TREK-1 channels, despite the basal current being much smaller. This is in contrast to the effect of FFA, which produces a significantly larger increase in current through loss of function TM4.6 (and TM2.6) channels compared to WT TREK-1 channels.

### Mutation G286R at TM4.6 of TREK-1 channels

**Figure 6:**
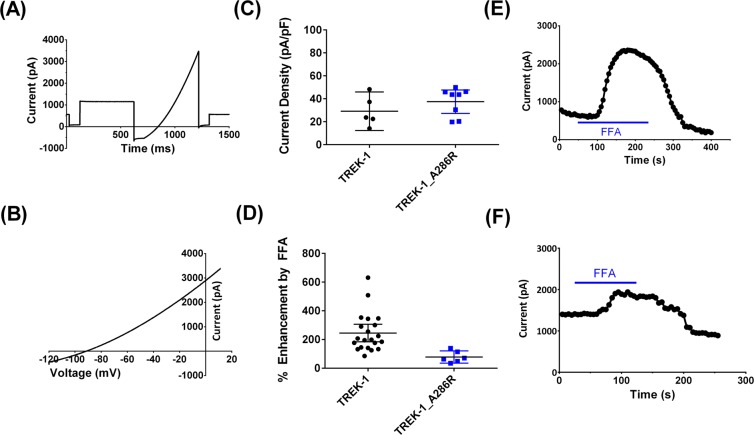
TM4.6 A286R mutant TREK-1 channels have reduced sensitivity to FFA. (**A**,**B**) Representative currents through TREK-1_A286R channels. (**C**) Current density for WT and TREK-1_A286R channels. There was no significant difference in current density between the two. (**D**) Percentage enhancement of WT and A286R channels by FFA. Percentage enhancement was significantly reduced for the mutant channel compared to WT (p = 0.006, t test). (**E**,**F**) Representative time course plots for enhancement by FFA of WT (**E**) and A286R (**F**) channels.

For TASK-3 K2P channels, mutation of the TM4.6 position to arginine (G236R) gives rise to KCNK9 imprinting syndrome^27,28^. For TASK-3 channels, this is a loss of function mutation with distinctive inwardly rectifying current^29^. Notably, current can be restored through these TM4.6 mutated TASK-3 channels by gain of function mutations (A237T) and, modestly, by FFA^29^. Mutating the equivalent TM4.6 residue in TREK-1 channels to arginine (G286R) has quite different effects to those seen for TASK-3 channels. Current amplitude is unaltered following this mutation and is not inwardly rectifying (Fig. 6A–C). However, the activation of the channel by FFA is significantly *reduced* compared to WT TREK-1 channels (Fig. 6D–F).

### Gain of function mutations restore current through TM4.6 mutated TREK-1 channels

**Figure 7:**
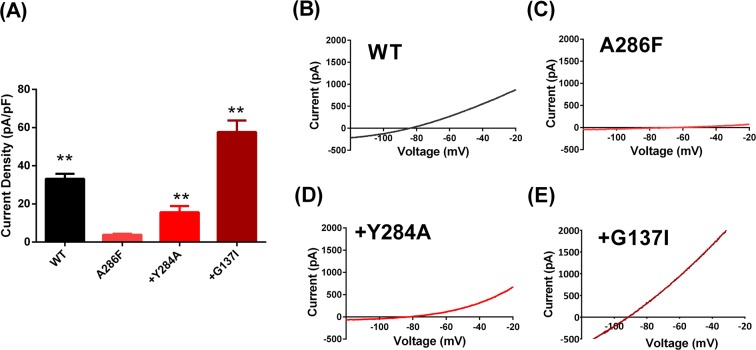
Gain of function mutations recover current through TM4.6 (A286F) mutated TREK-1 channels. (**A**) Histogram of outward currents measured as the difference current between that at −40 mV and −80 mV for WT TREK-1 and mutated TREK-1 channels (** = significantly different from TREK-1_A286F channels at <0.05 level, ANOVA followed by Tukey’s test). (**B**–**E**) Representative current-voltage relationships for WT and mutated TREK-1 channels.

Previous work has shown that gain of function mutations can restore, at least in part, current through reduced function variants of TREK-1 (e.g.^12^). Lolicato *et al*.^30^ have shown that the TM4 and TM2 helices contact each other along a hydrophobic interface. They identified several amino acids in each helix that make contact in WT TRAAK channels, including L156 (TM2.4), I159 (TM2.7) and F272 (TM4.5), all adjacent to the equivalent TM2.6 and TM4.6 residues in TRAAK. In the G124I gain of function mutation of TRAAK, the TM4 helices straighten compared to WT TRAAK, centred on the conserved TM4.1 glycine (G268) with the largest movement seen for F272 (TM4.5). Indeed, there is significant movement of both the TM2 and TM4 regions of the TRAAK channel in crystal structures of gain of function mutations, suggesting that these regions of the channel move when the channel is gated at the selectivity filter. Consistent with these observations on TRAAK channels, we have found that currents through loss of function TM4.6 TREK-1 (A286F) channels could be restored to 57.7 ± 6 pA/pF (n = 14) when the channels also carried the equivalent gain of function mutation, G137I, (Fig. 7A–C,E), which is also larger than WT current through TREK-1 channels. A second gain of function mutation (Y284A^12^) also gave currents that were significantly larger than seen with the A286F mutation alone (Fig. 7A–D).

### C286 crosslinking at TM4.6 hampers FFA regulation of TREK-1

Similar to the movement of TM4 seen for gain of function mutations, polymodal regulation at the C terminus is also thought to be transduced to the selectivity filter gate through movement of the TM4 helices (e.g.^23^). Furthermore, mutations which disrupt this gating pathway have been shown to reduce activation of the channel by BL-1249^16^.

MTS (MethaneThioSulfonate) cross-linking agents can be used to link two accessible cysteine residues in a protein by forming a disulphide bond with each of these residues. Since the TM4.6 residue (A286) is facing the pore, C286 mutated residues will be accessible from the intracellular side of the membrane. We hypothesized that TREK-1 A286C cross-linked residues might interfere with the movement of the TM4 helix that occurs when channels are gated open at the selectivity filter^17^ and, as a consequence, hamper regulation of the channel by FFA.

FFA (100 µM) enhanced the current through TREK-1 A286C channels by 235 ± 30% (n = 5). Furthermore, current through A286C mutated channels was similar in amplitude to that seen for WT TREK-1. This shows that the A286C mutation does not, in itself, change the TREK-1 channel properties nor diminish the channel sensitivity to FFA.

**Figure 8:**
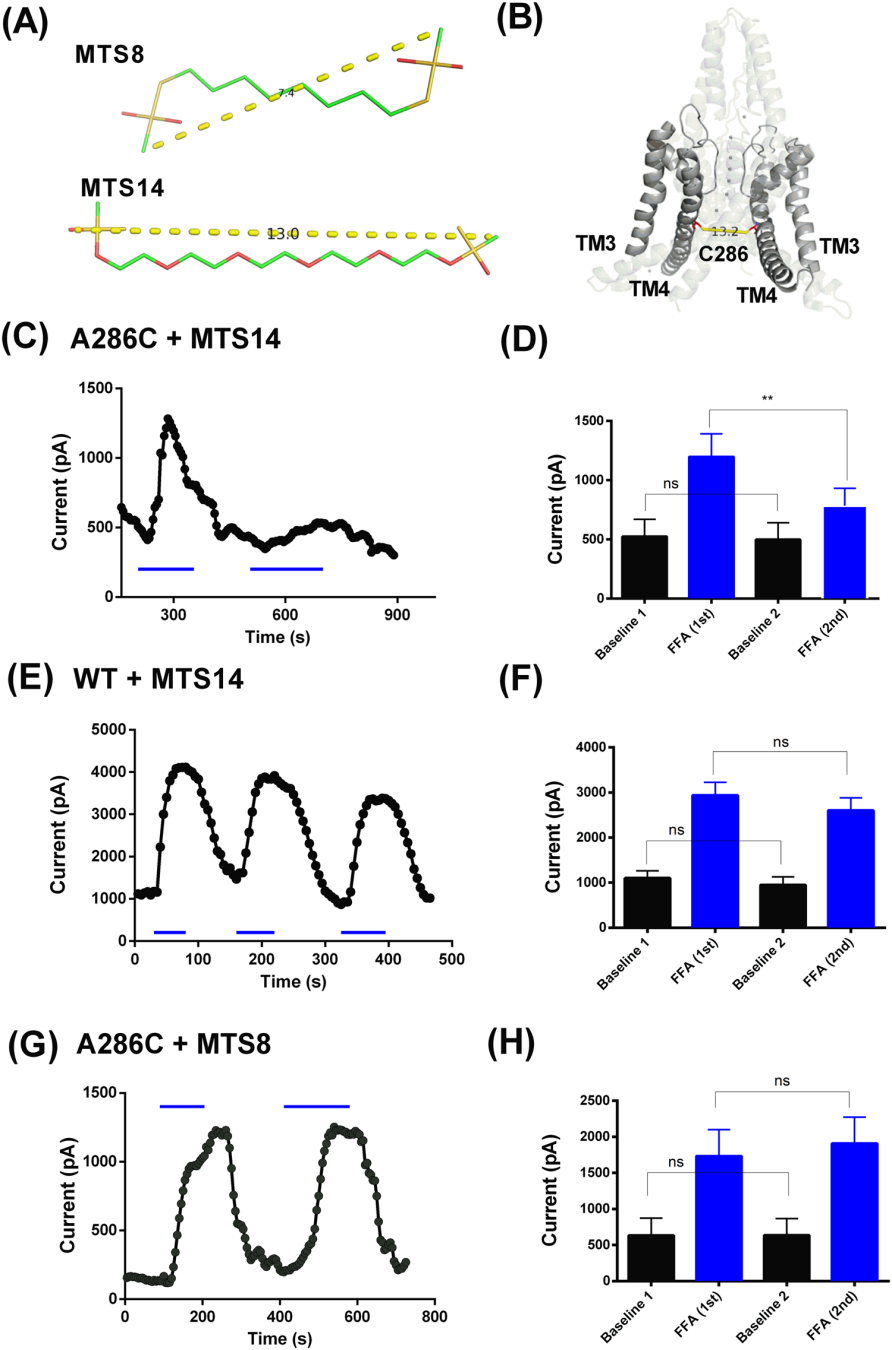
TM4.6 crosslinking hampers FFA regulation of TREK-1. (**A**) Molecular representation of MTS-8 and MTS-14. (**B**) Illustration of the TM3 and TM4 regions of TREK-1_ A286C structure created from the pdb file for the crystal structure of TREK-1^17^ (6GC6), indicating the distance between the C286 residues. (**C**) Representative time course for enhancement of TREK-1_A286C current by FFA (100 µM) in the presence of intracellular MTS-14. (**D**) Histogram of outward currents measured as the difference current between that at −40 mV and −80 mV for TREK-1_A286C channels before (black) and during (blue) consecutive applications of FFA (100 µM) in the presence of intracellular MTS-14 (** = significant difference between first and second applications of FFA at p = 0.002, paired t test). (**E**) Representative time course for enhancement of WT TREK-1 current by FFA (100 µM) in the presence of intracellular MTS-14. (**F**) Histogram of outward currents measured as the difference current between that at −40 mV and −80 mV for WT TREK-1 channels before (black) and during (blue) consecutive applications of FFA (100 µM) in the presence of intracellular MTS-14 (no significant difference between first and second applications of FFA at 0.05 level, paired t test). (**G**) Representative time course for enhancement of TREK-1_A286C current by FFA (100 µM) in the presence of intracellular MTS-8. (**H**) Histogram of outward currents measured as the difference current between that at −40 mV and −80 mV for TREK-1_A286C channels before (black) and during (blue) consecutive applications of FFA (100 µM) in the presence of intracellular MTS-8 (no significant difference between first and second applications of FFA at 0.05 level, paired t test).

We used two MTS crosslinking agents, MTS14-04-MTS (MTS14) and MTS-8-MTS (MTS8), the length of the linkers being 13 Å and 7.4 Å respectively (Fig. 8A). The length of MTS14 is close to the predicted distance between the C286 residues (13.6 Å), from a cysteine-substituted model created from the crystal structure of TREK-1^17^ (PDB: 6GC6, Fig. 8B), whilst the length of MTS8 is considerably smaller. Freshly made up MTS14 and MTS8 were added to the intracellular pipette solution immediately before whole-cell voltage-clamp recording and were allowed to dialyse into the cell for 5 mins before recording commenced. FFA was applied at least twice to each cell to allow a comparison before and after initial exposure to FFA. While FFA reversibly activated TREK-1 in each application, the current during the second FFA application was significantly reduced when compared to the first FFA application (Fig. 8C,D). The initial application of FFA on TREK-1 A286C enhanced current by 210 ± 83% (n = 6), whilst the second FFA application produced a significantly smaller increase (p < 0.03, one-sided paired t test) in TREK-1 A286C current of 112 ± 52% (n = 6) in the same cells. The response to the second application of FFA to TREK-1 A286C channels in the presence of MTS14 was also significantly smaller (p = 0.002, one-sided paired t test) when measured as the absolute current observed in the presence of FFA (Fig. 8D). Additionally, in contrast to control cells, the enhancement by FFA began to decline during the first application of FFA despite the continued presence of the compound (Fig. 8C). This indicates that MTS14 interacted with the mutated TREK-1 A286C and reduced the effect of FFA on the channel following multiple applications (or continuous application) of the compound. Importantly, the basal current was not significantly different before or after the first application of FFA (Fig. 8D) and was not significantly different to the seen with A286C in the presence of MTS8 (Fig. 8H).

WT TREK-1 currents were enhanced similarly by the first and second FFA applications in the presence of intracellular MTS14. The initial application of FFA on WT TREK-1 channels increased the current by 194 ± 58% (n = 7), whilst the second FFA application increased WT TREK-1 current by 208 ± 43% (n = 7) (Fig. 8E,F). The shorter length MTS compound, MTS8 was used as additional control. This experiment followed the same protocol as with MTS14 in TREK-1 WT and TREK-1 A286C. The first application of FFA on TREK-1 A286C increased the channel currents by 277 ± 134% (n = 3), whilst the second FFA application increased TREK-1 A286C current by 282 ± 110% (n = 3). Therefore, unlike MTS14, the MTS8 compound showed no effect on the FFA enhancement of TREK-1 A286C currents (see Fig. 8G,H).

Thus, cross-linking cysteine residues at position TM4.6 reduced the activation of TREK-1 channels by multiple applications of FFA.

## Discussion

We have shown that mutation of the TM2.6 and TM4.6 residues of TREK-1 to phenylalanine residues (G171F and A286F) substantially reduces current through TREK-1 channels and that this reduction in current can be reversed, pharmacologically, by FFA. Thus, depending on the substituted amino acid, mutation at this position can alter channel gating to either enhance^18^ or reduce channel current. It is unlikely that these mutations cause substantial alterations in trafficking of TREK-1 channels. Current through mutant channels (G171F and A286F) in presence of FFA are significantly larger than WT current suggesting that mutant channels are present at the membrane and available for regulation. Furthermore, Ben Soussia *et al*.^18^ showed that mutations of the G171 to different residues, namely G171D and G171N did not result in any alterations in expression despite marked increase in current (gain of function) produced by these mutations.

For at least some K2P channels, particularly those in the TREK family, it has been proposed that gating occurs via a single common gate near the selectivity filter, and that the primary activation gate seen in many other K channels^19,31^ is constituently open^22–24^. Both extracellular regulators such as protonation^26^ and intracellular modulators including heat, intracellular acidosis, and mechanical stress^32,33^ exert their effects by targeting this common TREK-1 gating mechanism.

Following publication of the original crystal structures of TWIK-1 and TRAAK^34,35^, a number of different structural forms of TREK channels have been identified, which correspond to different conformations of the channels^14,15,17,30,36^. Structures of gain of function mutations of TRAAK^30^ show straightening and tilting of TM4 and buckling of TM2 compared to the control conformation. In this study, the introduced loss of function mutations in the inner pore helix at TM2.6 and TM4.6 (A286F and G171F), were used to probe the conformational changes induced by both gain of function mutations and FFA in TREK-1 channels. The gain of function mutation G137I was able to fully reverse the current reduction caused by the introduction of A286F. This provides evidence to support movement of the TM4 region to gate the channel and is consistent with the crystal structure of Lolicato *et al*.^30^ for the equivalent TRAAK channel G124I mutant, where TM4 straightens compared to WT TREK-1. The largest movement between WT and G124I channels is seen for F272 (TM4.5), adjacent to TM4.6 and straightening of TM4 is centred on the conserved glycine at position 268 (TM4.1) in the mutated channels. Interestingly, the crystal structures of the two gain of function mutant TRAAK channels described by Lolicato *et al*.^30^ are different, which suggests that TREK channels can adopt a number of different conformations^37^ or even a continuum of conformations when moving between a fully closed state and a fully open state.

FFA also reversed the reduction of current seen with the G171F and A286F mutations. This suggests that FFA activation of TREK-1 may also induce movement at the region of the A286 and G171 residues in the TM2 and TM4 inner helices to restore conduction through the channel, in a similar manner to that observed with the gain of function mutations. Alternatively, FFA binding may act to bias the natural fluctuations between channel conformations and stabilize the channel in its active conformation.

Pope *et al*.^16^ showed that “triple glycine” C terminus mutants uncouple the C terminus from gating and blunt the response to the related NCA, BL-1249 (see also^38^) which suggests an interaction between NCAs and C terminus gating. Similarly, NCAs have a reduced effectiveness on TREK-1 channels which have gain of function mutations compared to WT channels^12^. These observations suggest that regulation by NCAs and regulation through movement of TM4 is linked. On the other hand, Schewe *et al*.^15^ showed that NCAs bypass specific gating mechanisms to act as “master keys” to open the channel, binding to sites immediately below the selectivity filter to increase K occupancy and open the filter gate.

In this study, substituting A286 in the TREK-1 pore to a cysteine (C), and crosslinking the A286C residues from the two TREK-1 subunits by an MTS reagent of appropriate length did not affect basal current amplitude but decreased the sustained activation by FFA or the response to repeated FFA applications. This may suggest that, in the presence of FFA, the channel favours a conformation that promotes C-C crosslinking at position TM4.6 or, less likely, linking of C286 with one of the three cysteine residues (C93, C159 and C259) present in WT TREK-1. From the structure of TREK-1, none of these are obviously accessible to C286 in terms of location or distance. C93 is located at the peak of the M1P1 loop, not close to C286. C159 is on TM2 behind the selectivity filter and C219 is near the middle of TM3. Alternatively, as more cross-linking develops with time following the beginning of whole-cell recording, FFA becomes less effective because as a result of cross-linking, movement of TM4 will be impeded and fluctuations between different confirmations reduced.

More generally, our data suggest a reciprocal interaction between binding of NCAs and movement of TM4 either by regulators which act on the C terminus of the channel or by gain of function mutations, with all converging on the selectivity filter gate. In support of this, mutation of either of the conserved threonine residues (T142 and T251) in the P1 and P2 pore loops blunts activation by FFA and BL-1249 (see also^15^). Mutation of the same threonine residues also blunts activation of TREK-1 by arachidonic acid and PIP_2_^21^.

There is evidence to suggest that 2-aminoethoxydiphenyl borate (2-APB), which shows some selectivity for TREK-2 over TREK-1 channels^39^, also requires movement of the TM4 segment to activate TREK-2^40^, whilst the dihydroacridine analogue, ML67-33, is proposed to activate TREK channels by interacting directly with the extracellular C-type gate^41^. Additionally, the pharmacological activators ML335 and ML402 have been shown to act as molecular wedges to stabilize the channel selectivity filter gate in its more active conformation through binding to a novel cryptic binding site adjacent to the selectivity filter^17^, distinct from the NCA binding site^15^.

Movement of the inner pore helices is a common feature of K channel gating. Translational and rotational movements of the TM2 pore helix of KcsA increases current flow through the channel^42^ with similar movements in the equivalent TM6 transmembrane helices proposed for allosteric coupling between the inner pore helix and the selectivity filter gate in the K_V_ potassium channel family (see^43^).

The same TM2.6 and TM4.6 inner pore helix residues of TRESK channels are large F residues. We have found that mutation of these amino acids to A (F125A_F352A), the complementary experiment to that on TREK-1 channels, gives significantly more current compared to WT TRESK channels. It has been proposed that this position forms a binding site for blockers targeting mouse TRESK channels^44,45^. Our data suggest, rather, that mutations at this position alter channel gating to occlude inhibitory drug action regardless of where on the channels the compounds bind (see also^38,46^). Thus functional current can be induced in TRESK channels and reduced in TREK-1 channels by suitable mutation of TM2.6 and TM4.6.

At the same position in the K2P channel TWIK-1, L146 (TM2.6) and L261 (TM4.6) line the narrowest point of the inner pore^47^ and the channel is primarily dewetted and non-conducting^47^. Hydrophilic substitutions of these amino acids (e.g. L146N) give a large increase in current density^18,47^.

In TASK-3 K2P channels, the G236R mutation at position TM4.6, gives rise to KCNK9 imprinting syndrome^27,28^. The introduced bulky and positively charged arginine leads to pronounced inward rectification of current through these mutated channels as well as a substantial reduction in current^29^, confirming the potential importance of this residue in influencing channel activity in K2P channels, generally. Interestingly, the equivalent TM4.6 arginine mutation in TREK-1 (A286R) did not affect current amplitude or rectification but did substantially reduce the degree to which current could be activated by FFA (Fig. 6).

There is increasing evidence in support of a role of TREK channels in nociceptive processing, which may offer hope of new therapeutic advances in the treatment of pain^9,10^. For example, TREK-1 channels are expressed in certain dorsal root ganglion neurons where they are co-localised with excitatory TRPV1 channels^48–50^. Furthermore, TREK-1, TRAAK and dual (TREK-1/TRAAK) knockout mice show increased sensitivity to painful heat sensations and a variety of other painful stimuli including inflammatory and mechanical stimuli^50,51^. It is suggested that the pain-relieving actions of morphine may be linked to activation of TREK-1 channels^52^. TREK-2 channels also contribute to the resting membrane potential of certain DRG neurons and, as such, these channels are proposed to limit signal transmission in pathological pain^53^.

The putative importance of TREK channels in pain suggests that compounds which enhance their activity, such as FFA described here, would be of considerable value as lead compounds for potential new analgesic agents targeting these channels. Pharmacological activation of postsynaptic TREK-1 channels would hyperpolarise the membrane of sensory neurons and depress neuronal activity in the pain pathway thus countering excitatory stimulation by noxious stimuli^9,12,54^. Future experiments, such as gene editing, which utilise the pharmacologically reversible loss of function mutations at positions TM2.6 and TM4.6 of TREK channels described here, will help to clarify the importance of TREK channels in pathophysiological conditions such as pain and depression. These will complement experiments with gain of function mutations at the same position, as proposed by Ben Soussia *et al*.^18^.

## Methods

Most of the methods used in this study have been used and described by us previously^12,29,38^. A summary of the methods used and variations specific to this study are given below.

### Cell culture

tsA201 cells (ECACC, Sigma-Aldrich, Gillingham, Dorset, UK), were grown in a monolayer tissue culture flask maintained in a growth medium which was composed of 88% minimum essential media with Earle’s salts and 2mM L-glutamine, 10% of heat-inactivated fetal bovine serum, 1% penicillin (10,000 units ml^−1^) and streptomycin (10 mg ml^−1^), and 1% non-essential amino acids. The cells were placed in an incubator at 37 °C with a humidified atmosphere of 95% oxygen and 5% carbon dioxide. After 2 or 3 days, when the cells were 70 to 90% confluent, they were split and resuspended in a 4 well plate containing 13 mm diameter cover slips (poly-D-lysine coated) in 0.5 ml of media, ready to be transfected the next day.

### Transfection

For the electrophysiological experiments, pcDNA3.1 vector was cloned with the gene of interest (hTREK-1 or hTRESK wild-type or mutated) and a similar vector containing GFP were incorporated into the cells (0.5 µg per well for each plasmid) using the calcium phosphate method. The cells were incubated for 6–10 hours with the transfection solution. Then, cells were washed using a phosphate buffered saline solution (PBS), and new media was added to each well. The cells were used for experiments after 12–24 hours.

### Mutations

Point mutations were introduced by site-directed mutagenesis into TREK-1 or TRESK channels using the Quikchange kit (Agilent Technologies). A pair of short (25–35 bases) complementary oligonucleotide primers, incorporating the intended mutation, were synthesized (Eurofins MWG Operon, Ebersberg, Germany). Mutant DNA constructs were sequenced (Eurofins MWG Operon) to confirm the introduction of the correct mutated bases.

### Whole cell patch clamp electrophysiology

Currents were recorded using the whole cell patch clamp in a voltage clamp configuration in tsA201 cells transiently transfected with the channel of interest. The cover slip with the cells was placed in a recording chamber filled with an external medium composed of 145 mM, NaCl, 2.5 mM KCl, 3 mM MgCl_2_, 1 mM CaCl_2_ and 10 mM HEPES (pH to 7.4, using NaOH). The internal medium used in the glass pipette comprised 150 mM KCl, 3 mM MgCl_2_, 5 mM EGTA and 10 mM HEPES (pH to 7.4, using KOH). Modulatory compounds were applied by bath perfusion. Complete exchange of bath solution occurred within 100–120 s. All data were collected at room temperature (19–22 °C). The transfected cells were detected using a fluorescent microscope with UV light. Cells were voltage-clamped using an Axopatch 1D or Axopatch 200B amplifier (Molecular Devices, Sunnyvale, CA, USA) and low pass filtered at 5 kHz before sampling (2–10 kHz) and online capture.

In order to study the potassium leak current, a “step-ramp” voltage protocol was used. For the step component of the protocol, cells were hyperpolarised from a holding voltage of −60 mV to −80 mV for 100 ms then stepped to −40 mV for 500 ms. For the ramp, cells were then stepped to −120 mV for 100 ms, followed by a 500 ms voltage ramp to +20 mV and a step back to −80 mV for another 100 ms, before being returned to the holding voltage of −60 mV. This protocol was composed of sweeps lasting 1.5 seconds (including sampling at the holding voltage) and was repeated once every 5 seconds (see^12^). For analysis of outward current, we measured the current difference between the −80 mV and −40 mV steps. For comparisons between different cells (such as between WT and mutated channels) current density was used (pA/pF). For comparisons within cells (such as before and after a drug treatment) either change in absolute current or % change was measured. The current-voltage graphs were obtained from the ramp change in voltage between −120 mV and +20 mV. The currents obtained with the imposed voltage protocol were recorded and analysed using pCLAMP 10.2 software and Microsoft Excel.

### Data analysis

Data were expressed as mean ± standard error of the mean (SEM) and ‘n’ represents the number of cells used for the experiment. The statistical analyses used either the Student t-test (both paired and unpaired) or a one way ANOVA with the post-hoc Dunnett’s or Tukey’s multiple comparisons tests, using GraphPad Prism 6.02. For the t-test, the differences between means were considered as significant for p < 0.05 for the purposes of data interpretation and discussion, but precise p values are given in the text and figure legends. Similarly, for the Dunnett’s or Tukey’s tests, for the purposes of data interpretation and discussion, data were considered significantly different at the <0.05 level (confidence interval >95% for the difference between the two compared means) but precise p values are given in the text and figure legends.

### Chemicals

MTS compounds were purchased from Toronto Research Chemicals, Canada. 3,6,9,12-Tetraoxatetra-decane-1, 14-diyl-bis-methanethiosulfonate “MTS14” (MTS-14-O4-MTS) and 1,8-Octadiyl Bismethanethiosulfonate “MTS8” (MTS-8-MTS) were made up in DMSO and the aliquoted stock solutions stored at −20 °C until use. 0.5 mM of MTS14 or MTS8 were made fresh by dilutions into the intracellular solution immediately prior to each individual recording. All other fine chemicals were purchased from Sigma-Aldrich, Gillingham, Dorset, UK. Flufenamic acid (FFA) stock (10 mM) was made up in ethanol and diluted fresh in external solution before use (pH adjusted to 7.4).

### Homology modelling

The crystal structure of hTREK-1 was PDB ID: 6GC6 (updated from^17^). The homology model of TRESK was created using Modeller 9v8^55^, with a TRAAK structure (PDB ID: 3UM7) as a template. ClustalW^56^ was used to align channel sequences.

## Data Availability

The datasets generated during and/or analysed during the current study are available from the corresponding author on reasonable request.
